# Variable Offset Computation Space for Automatic Cooling Dimensioning

**DOI:** 10.3390/polym14040762

**Published:** 2022-02-16

**Authors:** Christian Hopmann, Daniel Colin Fritsche, Tobias Hohlweck, Julius Nehring-Wirxel

**Affiliations:** 1 Institute for Plastics Processing (IKV) in Industry and Craft at RWTH Aachen University, 52070 Aachen, Germany; office@ikv.rwth-aachen.de (C.H.); tobias.hohlweck@rwth-aachen.de (T.H.); 2 Visual Computing Institute, RWTH Aachen University, 52070 Aachen, Germany; sekretariati8@informatik.rwth-aachen.de

**Keywords:** variable offset, thermal simulation, cooling channel, medial axis, mold design

## Abstract

The injection mold is one of the most important elements for the part precision of this important mass production process. The thermal mold design is realized by cooling channels around the cavity and poses as a decisive factor for the part quality. Thus, the objective but specific design of the cooling channel layout is crucial for a reproducible part with high-dimensional accuracy in production. Consequently, knowledge-based and automated methods are used to create the optimal heat management in the mold. One of these methods is the inverse thermal mold design, which uses a specific calculation space. The geometric boundary conditions of the optimization algorithm influence the resulting thermal balance within the mold. As the calculation area in the form of an offset around the molded part is one of these boundary conditions, its influence on the optimization result is determined. The thermal optimizations show a dependency on different offset shapes due to the offset thickness and coalescence of concave geometries. An algorithm is developed to generate an offset for this thermal mold design methodology considering the identified influences. Hence, a reproducible and adaptive offset is generated automatically for a complex geometry, and the quality function result improves by 43% in this example.

## 1. Introduction

The mold of the injection molding process provides the boundary conditions for the physical actions taking place from the injection phase until ejection of the molded part. Material-related component shrinkage is one of the greatest challenges of modern plastics processing and poses a challenge for high geometric and shape part precision. This accounts especially for injection molded components, which are characterized by a high functional density and increasingly complex component geometries. From the surface and geometry of the cavity to the heat transfer coefficient between mold and melt as well as the arrangement of cooling channels, the design, chosen material and mold manufacturing methods have a significant influence on the physical properties of the plastic product [1]. These dependencies within the mold and part design are additionally affected by multiple influencing factors such as processing conditions, humidity, post-shrinkage conditions, etc. [2] These effects superimpose each other, which leads to a locally varying part shrinkage that needs to be anticipated during the mold design phase. The overall challenge is that customers often have high demands on surface appearance and geometric stability. Warpage occurring in plastic components is a critical problem in manufacturing, often leading to deviations from shape and position tolerances or to reworking of the molded part and the associated injection mold. The current standard in thermal mold design is process simulation using commercially available software. Based on the results of the simulation, the temperature control layout is adjusted, and the success of the adjustment is checked in a new iteration of the simulation. This process is repeated until the process and quality parameters are within a satisfactory range. Therefore, especially temperature and pressure gradients during cooling have to be considered to predict and compensate warpage due to inhomogeneous shrinkage.

Many research groups work in the automatic generation of cooling channels. An extensive literature review has recently been performed by Feng et al. [3]. Accordingly, the scientific approaches are clustered into five categories:

- Experiment-based design;
- Design and optimization based on the conformal cooling line;
- Optimization using expert algorithms;
- Modular/parametrical design of conformal cooling channels;
- Solid modelling based on topology optimization.

The first category is a hands-on approach, where different designs are created via experience and then validated via simulations [4,5,6]. In the second category, cooling channels are geometrically optimized by keeping a constant distance to the molded part. This is a very interesting approach, as it is very quick in terms of calculation time [7,8]. Nevertheless, it does not consider different wall thicknesses or the increased cooling demand for complex geometries. Several sophisticated mathematical approaches can be grouped under the category “Optimization using expert algorithm” [9,10]. However, expert knowledge is necessary to be able to process these algorithms. Modular design of conformal cooling channels is a category where the shape of the cooling channels is parameterized (e.g., the diameter and/or the length) [11,12]. These approaches generate very quick results, but they do not necessarily cover all possible solutions and therefore do not guarantee an absolute minimum of warpage. In the category of topology optimization, several approaches are summed up, which improve e.g., the local heat conductance of the steel to improve the heat balance of the mold. These approaches take the tempering channel design as a given constant [13,14]. As it can be seen, the different approaches all have their advantages and disadvantages.

The Institute for Plastic Processing in Industry and Craft at the RWTH Aachen University (IKV) researches the method of the inverse thermal mold design. This method falls into the category of *optimization using expert algorithms*, but it places low demands on the designer in a pure application. The designer does not have to specify any parameters for the calculation of the cooling channel layout other than the planned cooling time and the targeted demolding temperature. The method calculates an optimal heat balance in the injection mold independently of an existing cooling channel system (see Section 2.1). Thermal optimization and calculation of the heat flow during cooling takes places within a certain geometry around the investigated part, which defines the calculation area and degrees of freedom. This offset around the molded part has an influence on the possible optimization quality due to the limited position of the optimization parameters. The contour and thickness of the offset can be set and, therefore, change the number of degrees of freedom of the optimizer and the distance to part or individual sections of it. Consequently, the influence of this contour on the resulting temperature distribution and cooling rate of the optimization is further analyzed. Based on this, an algorithm was developed that takes into account the geometric properties of the molded part. In this work, existing concepts from the geometry processing field are adapted and combined to a robust and automated pipeline that takes an input model and produces a variable offset that is then used to define the domain for the thermal optimization. In previous work, the offset domain was either computed with constant offset, or it had do be manually generated. Therefore, the methodology of the inverse thermal mold design is further developed. Research has shown the benefit of an automated derivation of cooling channels from the optimized heat balance [15], the evaluation of the local cooling rate and freeze time during optimization [16], and canceling out the influence of the mesh on the evaluated temperature distribution [17]. For this reason, the research in this work focuses on the contour around the analyzed part, which has not yet been studied. A step toward fully automated generation of cooling channels is contributed.

## 2. Related Work

### 2.1. Methodology of an Inverse Thermal Mold Design

This work uses an optimization algorithm to calculate a thermal balance within the mold, which provokes a cooling of the molded part with minimized warpage. Within the optimization of the inverse mold design, the thermal boundary conditions in the mold are calculated in such a way that a thermally optimal molded part is produced at the end of the cooling phase with regard to the material models and modeling used. This methodology is based on an approach by Agazzi et al. [7], was extended by Nikoleizig [18], and is further researched at IKV [17,19,20]. In this approach, a thermal optimization of the injection mold is carried out on the basis of a quality functional, which objectively quantifies the molded part quality after cooling in the injection mold. Agazzi et al. [7] use a purely thermal approach to evaluate the molded part quality, which is supplemented by further material-specific and numeric aspects. Extensive research in different fields of this methodology has been performed to design reproducible and objective cooling channels to compensate the need for lower warpage and higher precision.

Before the optimization step, a contour is created in the form of an offset of the part. This geometry maps the steel tool around the molded part. This has the advantage of a smaller calculation space than compared to the whole mold, which is multiple times bigger. Additionally, the contour’s surface is the location of the temperature values, which are varied by the optimizer. These temperatures are the optimization parameters that result in the optimized thermal balance.

**Figure 1:**
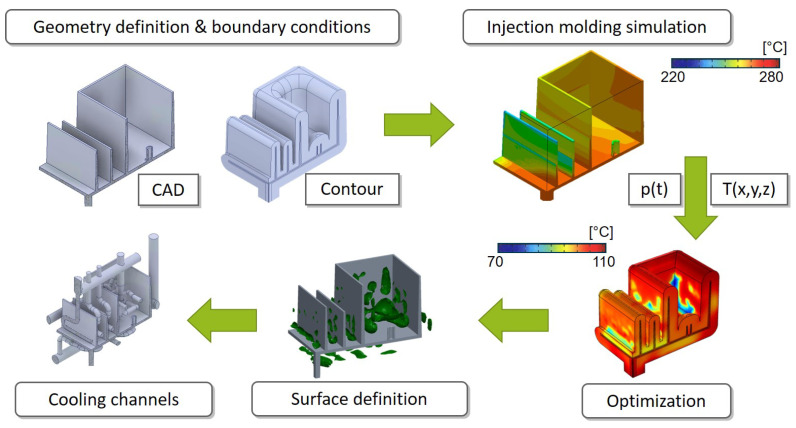
Overview of the inverse thermal mold design procedure.

The used routine for the thermal cooling channel design used in this research is based on the further development of Hohlweck [16]. Figure 1 shows that the generation of the offset is the first step within this method. Therefore, its influence affects the entire following procedure.

Once a suitable offset is created, an injection molding simulation is carried out using commercial models and software. This simulation depicts solely a temperature distribution after multiple cycles without any cooling channels within the mold. This allows a location-dependent temperature distribution to be quickly calculated for the molded part and the offset as a starting condition for the following optimization. Additionally, a time-dependent pressure distribution can be imported from the injection molding simulation to consider the pressure dependencies of the thermal properties and pvT (pressure, specific volume, temperature) behavior. These starting and boundary conditions are implemented in *Comsol Multiphysics*, *Comsol AB*, *Stockholm*, *Sweden* for the optimization step. They are particularly important because the approach of Agazzi et al. [7] is executed with gradient-based algorithms. These algorithms strongly depend on initial values and the later introduced quality function [21]. Starting from these calculated initial conditions, iterative convergence to the nearest optimum is performed.

The optimization deals with the boundary value problem of inverse thermal heat transfer [22]. The boundary values have to be calculated for the desired or targeted temperature distribution. Therefore, the temperature on the offset’s contour and the resulting heat conduction is calculated so that the temperature of the molded part during cooling is optimized. A quality function is necessary to evaluate the thermal condition in the molded part with the aim of minimizing distortion. The used quality function considers the influence of the temperature distribution as in [7] and the cooling rate to evaluate the morphology of the cooled polymer. In general, injection molded parts experience different cooling rates over the wall thickness. Close to the cavity wall, a high cooling rate can be observed due to the contact of the hot melt with the cold mold. In the middle of the part, the cooling rate is significantly lower due to the isolation of the plastic [2]. As this physical effect cannot be changed, it is important to generate homogeneous properties inside constant layers around the midplane of the part geometry. If the properties are not symmetric around the midplane, a lever effect is generated, and warpage can be expected. These aspects lead to the newly formulated quality function [17]:

(1) $$\begin{array}{c} Q\left(T_{KK_{0}}\right)=\sum_{i=1}^{m}(\frac{T_{ejec}-T_{P}\left(\vec{x_{i}},t_{c};T_{KK_{0}}\right)}{T_{ejec}}{)}^{2}\cdot \frac{A_{elem,i}}{A_{tot,1}}\cdot w_{i}+\\ \sum_{j=1}^{k}((\frac{\bar{\dot{T}\left(T\right)}-\dot{T}\left(\vec{x_{j}},T_{t}\right)}{\bar{\dot{T}}}{)}^{2}+\left(\frac{{\bar{t}}_{t}-t_{t}\left(\vec{x_{j}},T_{t}\right)}{{\bar{t}}_{t}}\right){)}^{2}\cdot \frac{A_{elem,j}}{A_{tot,2}}\cdot w_{j}. \end{array}$$

The first term of the equation is similar to the quality function from Nikoleizig [18]. It evaluates the temperature difference in the part $T_{P}$ to the demolding temperature $T_{ejec}$ at the end of cooling $t_{c}$. In addition, the term is normalized based on the demolding temperature $T_{ejec}$. Furthermore, the mesh influence is considered by the variable $A_{elem,i}$. This fraction ensures that every temperature node is only considered by the share of its respective element on the whole evaluation surface or volume $A_{tot,1}$.

**Figure 2:**
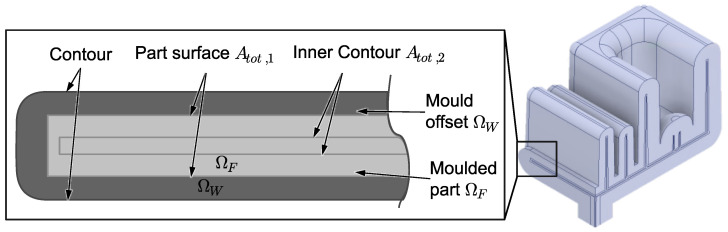
Detailed view of the domains and surfaces used for the optimization.

The second row of the equation evaluates the morphology of the molded part. The first term adds up the differences of the local cooling rates $\dot{T}$ compared to an averaged cooling rate $\bar{\dot{T}}$. The second term evaluates the solidification time $t_{t}$. Additionally, each term is weighted with the weight factors $w_{i}$ and $w_{j}$. These two terms become very small in the case of a homogeneous cooling rate at a similar solidification time. Both terms are evaluated on the $A_{tot,2}$ (Figure 2, left). $A_{tot,1}$ corresponds to the part surface. $A_{tot,2}$ is an offset surface inside the part. The inner offset is necessary, because at the interface of mold and melt, different cooling rates appear in steel and plastic, which lead to numerical instabilities in a gradient-based optimization. Based on the evaluation on these two surfaces, the optimization algorithm subtracts a temperature distribution on the mold surface such that the shown function becomes minimal.

An isosurface can be extracted from the optimal thermal heat balance in the following. This isosurface represents the position with the given heat flux that can be provided by cooling channels at this position. Along these isosurfaces, a developed algorithm approximates the segments of cooling channels and creates connections between them [15,16]. By taking into account obstacles such as ejectors, cavity, and parting plane and smoothing for a flow-optimized course, the calculated cooling channel system can be used practically.

### 2.2. Offsets

There is extensive literature on how to compute offsets from triangle meshes with constant offset diameters [23,24,25,26,27,28,29]. The ideal mesh offset is defined as the surface that has a constant shortest distance to the input surface at every point. This is equivalent to the *Minkowski* sum of the input manifold and a sphere with the chosen constant offset radius [23]. Given two input objects *P* and *Q*, their *Minkowski* sum is given by M(P,Q)={p+q|p∈P,q∈Q}. For the offset, imagine that the sphere’s center is moved to every surface point of the input mesh.

The approaches to compute constant radius offsets can be separated into three groups, including some hybrid approaches: Mesh Boolean-based, surface-based, and volumetric. Mesh Boolean-based approaches compute the *Minkowski* sum of the input object and a sphere. This can be solved by separating the sum into easier to compute primitives [28,29]: a sphere for each vertex, a cylinder for each edge, and a prism for each triangle. The spheres and the cylinders each have the constant offset as radius, while the prisms have the height of the offset and the triangle at the base. Unfortunately, these approaches inherit all the issues of Mesh Booleans in terms of precision, performance, and stability, while quickly creating highly complex problems at the same time [23].

Surface-based approaches usually try to directly apply offsets to input primitives [24,26,30]. This means that either all vertices or all faces are moved in the normal direction by the constant offset. For example, Kim et al. [24] add multiple vertices for each vertex to create a smooth offset. They do not resolve self-intersection in concave regions, as that is handled later by their NC tool-path generation application. On the other hand, Jung et al. [27] aim to efficiently eliminate all self-intersection from a raw offset mesh.

Finally, volumetric approaches evaluate the output surface in a grid and use isosurface extraction to extract the final offset mesh. The simplest candidate of volumetric approaches evaluates the signed distance to the input mesh in a regular grid and then uses an isosurface extraction algorithm such as marching cubes [31] to extract the boundary mesh at the desired offset radius. The difficulty lies in circumventing the $O(n^{3})$ complexity of the regular grid as well as avoiding aliasing effects caused by the grid. More sophisticated volumetric approaches such as [25] use an adaptive octree and add additional per octree cell data such as local normal estimates that allow the creation of higher-quality offset meshes.

#### Variable Offsets

While most previous work was done on algorithms to extract offset surfaces with a constant distance to the input mesh, there is little work on variable offset surfaces. The definition for a variable offset varies vastly: Musialski et al. [32] aim to change a shape by generating offset vectors that, when applied to the input positions, assure that the shape fulfills specific properties, such as e.g., a desired barycenter to assure the manufactured part swims with a fixed upright orientation while minimizing the geometric deviation from the original input part. To avoid self-intersections, they prohibit offset vectors from crossing estimates of the medial axis. Ross et al. [33] propose a variable face-offset based method: Each face is shifted by its own offset distance in normal direction. Their algorithm produces one, not necessarily triangular, output face per input face. Woerl et al. [34] offer a solution to render variable offsets where a radius is assigned to each vertex. Every point on each triangle is also assigned an offset radius by linear interpolation of the triangles’ three vertex radii. Similarly to some constant offset approaches, this allows them to separate the offset into three primitives: a sphere for each vertex, a cone frustrum for each edge, and an offset triangle that tangentially touches the three offset spheres. They apply ray tracing to evaluate the offsets.

The approach of this work uses a similar offset definition as Woerl et al. [34]: the desired offset is defined for discrete values on the input surface. Instead of a radius for each input vertex, the input surface is rasterized, and a radius is defined for each rasterized sample point. Then, each sample point and its radius define an offset sphere. Then, the envelope surrounding the input is efficiently evaluated as union of all spheres using a regular volumetric voxel grid. Similarly to Musialski et al. [32], these offsets are prevented to grow across the medial axis, although for different reasons that are explained in Section 5.2. The offset radii are generated by first evaluating the local thickness for each rasterized sample and then mapping the thickness to a radius. The optimal mapping is determined experimentally.

## 3. Creation of Offsets for Complex Molded Parts

The offset is created manually, which is a time-consuming and error-prone process for complex geometries. It is not viable for complex geometries with e.g., openings, narrow ribs, snap hooks, etc., which corresponds to the real requirements of injection-molded components. Comercial CAD software often provides tools for an automated offset orthogonal to the selected surfaces. However, this method leads to a larger and unrelated distance between the part and the offset at the edges. Even the rounding of edges and corners is only a limited remedy, as, for example, the maximum offset of inner radii is determined by their magnitude or the combination of several complex surfaces leads to geometric defects.

**Figure 3:**
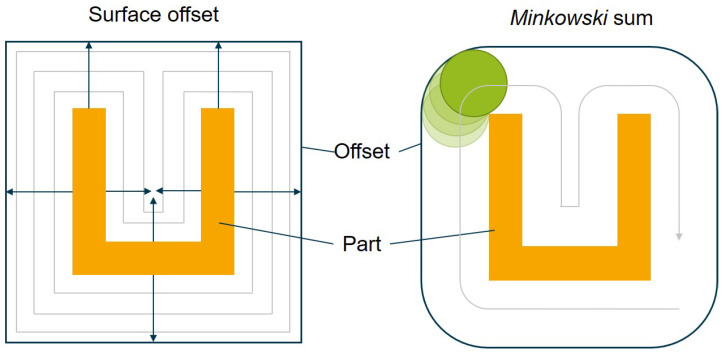
Generation of a contour with an orthogonal surface offset (**left**) and the *Minkowski* sum (**right**).

There are algorithms that use the *Minkowski* sum and can create a constant distance from the part geometry [35]. The aspects of each of the available methods are illustrated in Figure 3 using the cross-section of a U-shaped molding. Additionally, an inner offset of the part is created as a special area for evaluation during optimization.

**Figure 4:**
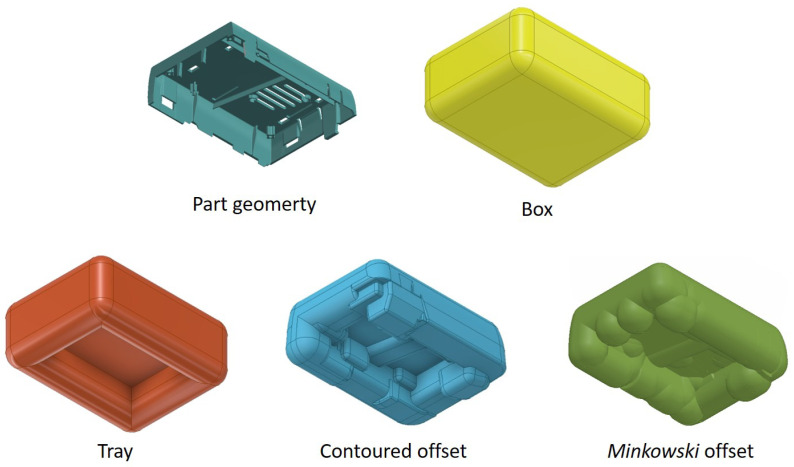
Investigated complex and simplified types of a contour for a manifold geometry. The geometric complexity increases from the box offset to the tray, to the contoured offset, and the *Minkowski* offset.

As the creation of contours with a constant distance to complex parts poses difficulties, the use of simplified contours is investigated. A case for a single-board computer (SBC) with small and complex elements such as ribs, screw domes, and snap hooks poses as molded part geometry. A minimal offset thickness of 10 mm is used for the investigation, but for the simplified contours, it is larger for various sections. In addition to the methods described above for creating a contour, a simple geometry in the form of a box and shell-shaped contour is created (Figure 4).

**Figure 5:**
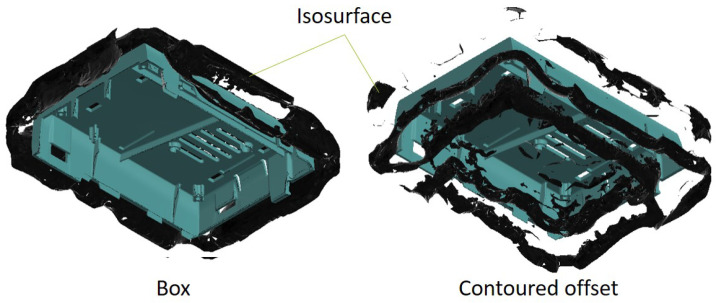
Resulting isosurfaces represeting the location of a certain heat flow for a box-shaped offset (**left**) and a contoured offset (**right**).

As a result, the isosurfaces, which indicate the position of the heat flow to be applied in the form of the cooling channels, are compared. With the box contour, the isosurfaces are evenly distributed around the part and have an approximately constant distance to it, whereas using the contoured offset results in a more specific isosurface (Figure 5). Therefore, a better result is expected for an offset that considers the source geometry and enables the derivation of specific cooling channel layouts.

## 4. Investigation of the Influence of the Offset on the Calculated Optimum

Since different creation methods of contours for the optimization step show various results for the same part and process conditions, the influence of an adaptive wall thickness is further investigated. Different factors determine the chosen size of the offset thickness. The outer offset of the molded part poses as a limiting factor as it limits the isosurfaces that are extracted. A big offset leads to a larger calculation space and can determine a larger portion of the needed heat flux position. Depending on the ejection time, ejection temperature, cooling fluid temperature, and the part’s geometry, a certain offset thickness is necessary to display enough isosurfaces to derive a channel layout. Accordingly, a large offset is advantageous. However, large offset thicknesses provoke high-temperature gradients on the offset contour to still achieve locally differing heat flows at the cavity. In analogy to the cooling error in injection molding molds, a large distance between the cooling channels and cavity causes a more homogeneous heat flow at the cavity [36]. Equation (2) shows the correlation between the cooling error *j* (j>0,1) and distance *l* between the cavity and cooling channel for a plate geometry of the width *b* and the *Biot* number Bi. It is used to calculate the cooling channel position to achieve a homogeneous heat flow over the cavity interface.

(2) $$j=2.4\cdot Bi^{0.22}\cdot {\left(\frac{b}{l}\right)}^{2.8\cdot ln(b/l)}.$$

Therefore, a strictly monotonically falling correlation of the cooling error and distance exists. A higher homogeneity of the heat flow is advantageous for parts with a constant wall thickness, but not for the optimization, which involves locally adapted heat flows for complex parts with different wall thicknesses. The superposition of the different heat flows set on the offset contour during optimization weakens the thermal flow resolution at the cavity. Consequently, the optimization algorithm sets up unrealistically high-temperature gradients on the contour to achieve the targeted heat flux at the cavity. These temperatures exceed the range for valid material parameters within the simulation.

### 4.1. Design of Experiment

The discussed influences (see Section 4) of the offset on the quality function are analyzed. The quality function is used to evaluate the influence of the offset, since it has been experimentally and simulatively validated that minimizing the function’s result causes more dimensionally accurate parts [15,16,18,19].

A simulative parameter analysis is conducted using a U-shaped part to investigate the effect of the offset thickness. A plain geometry allows an easier evaluation of the results by excluding complex thermal interactions and a simple manual offset generation as well as fast computing times. It has a height and depth of 50 mm and a distance of 60 mm between the flanks. Fourteen offset thicknesses from 6 to 15 mm are manually created for seven U-shaped parts each with wall thicknesses of 1 to 6 mm. The initial conditions for the various parts are calculated individually by a filling simulation with Sigmasoft from SIGMA Engineering GmbH, Aachen.

**Figure 6:**
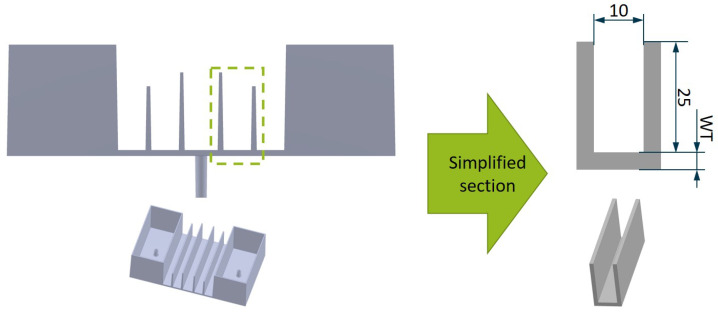
Pratical injection molding part (Complex-box) and the simplified section for further analysis of the offset geometry.

The effect of the coalescence is investigated with an adapted part geometry due the results described in Section 4.2. In order to create a heat accumulation in the mold and to provoke a necessary local heat flow, smaller distances between the elements of the geometry are realized. It is a section of the molded part (Complex-box) in Figure 6 that has been used in former research of the inverse thermal mold design [17]. The section represents plastic ribs with a practical dimensions such as the height and distance between them. Due to the practical origin of the geometry and the lower volume ratio of mold and cavity, a greater influence is expected from changes to the contour. The rib base with a high cooling demand is also less accessible due to the relatively large distance to the contour surface compared to the distance of the ribs.

**Figure 7:**
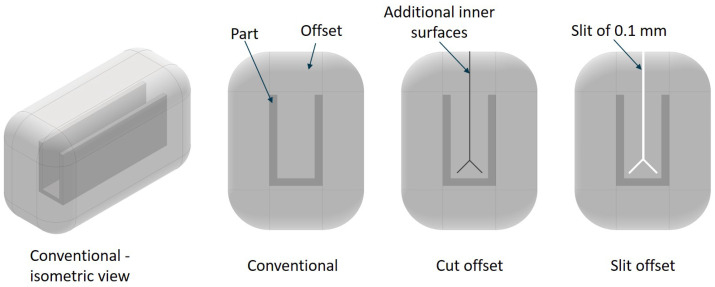
Analysed offset geometries for the evaluation of the influence of the medial axis on the optimization result.

As geometric compositions such as two narrow ribs have a high distance between the rib base and contour as soon as the offset thickness exceeds half of the rib distance, an additional surface is used and defined as an optimization area. The position of the additional surface is the medial axis. It represents all points that have more than one closest point on the object’s boundary (see Section 5.2). Three offsets of the geometry in Figure 6 with a wall thickness (*WT*) of 2 mm are examined. The conventional offset, one with an extra surface along the medial axis within the closed volume and one with a 0.1 mm thick slit along the medial axis, are compared in the thermal optimization (see Figure 7).

### 4.2. Influence of the Offset Thickness

**Figure 8:**
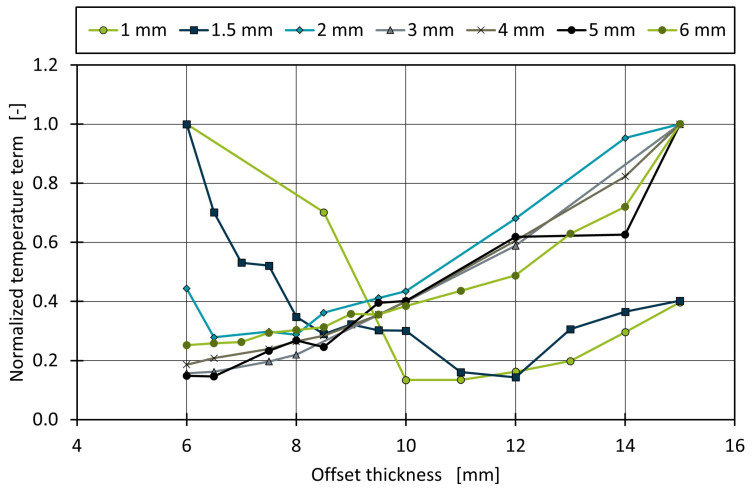
Normalized results of the temperature term for different wall and offset thicknesses.

The offset thickness is analyzed, and different results of the temperature distribution in the mold and quality function are examined. The resulting values of the quality function differ not only due to the offset thickness but due to the different wall thicknesses used. This results in a disproportionately higher temperature variation due to low thermal conductivity of the plastic. For a better comparison, the normalized results of the temperature term within Equation (1) are shown in Figure 8.

The two thinnest geometries show a minimum quality function value at 10–12 mm offset thickness and therefore indicate an optimal offset thickness. However, the simulation results for higher wall thicknesses show a steady increase of the quality function with higher offset thickness. An optimum could exist for smaller offset thicknesses, but this is not practical, as the calculation area would be too small for the subsequent approximation of the cooling channels. This result suggests an offset thickness of 8 or 10 mm for thin parts and the smallest possible offset thickness for thicker parts. With the right choice of the offset, the temperature distribution rating of the optimized heat balance within the mold decreases by 80%. The morphological term of Equation (1) does not show a significant influence of the offset thickness. This might be due to the fact that a geometry was chosen that has a wide basic geometry compared to the wall thickness of the part. Consequently, a high amount of steel is around the cavity compared to the mass accumulation at the edges. No critical heat spots at the edges of the U-shaped geometry occur, and therefore, the cooling rate is homogeneous.

### 4.3. Influence of an Extended Optimization Surface

**Figure 9:**
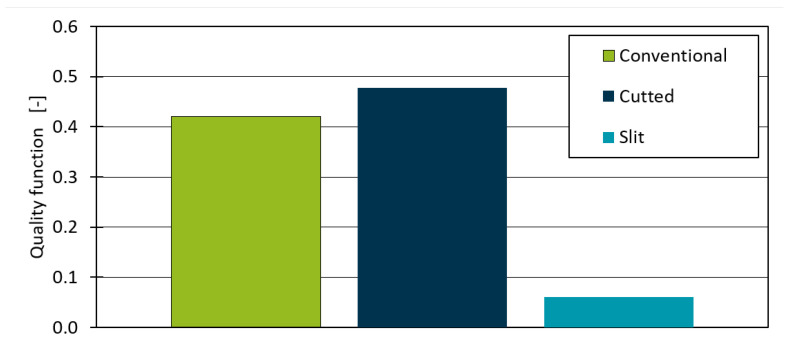
Results of the quality function for different designs of the medial axis in the offset.

The minimal value of Equation (1) after the last iteration of the optimization is depicted for each geometry in Figure 9. There is no significant difference between the conventional and extra surface geometry. As additional optimization surfaces are enclosed, no variation on this surface can be made. Otherwise, the thermal analysis would be overdetermined, or a heat source or sink is necessary. The small difference is explained by the differing mesh due to the differing geometric boundary conditions. However, the total result for the slit geometry is significantly smaller than the conventional contour. Due to the slit, the temperature on its surface can be varied by the optimization algorithm. Therefore, a more local temperature gradient at the rib base is applied, which supplies the necessary heat flow for a homogeneous temperature distribution, cooling rate, and freeze time. As the quality function is validated in former work a lower warpage can be expected when the optimized thermal balance is derived into an actual cooling channel design.

Consequently, an algorithm is developed to generate the contour for the methodology of inverse thermal mold design, which considers the shape of the part, its local wall thickness, and isolated sections with a high distance to the optimization surface.

## 5. Variable Offsets

**Figure 10:**
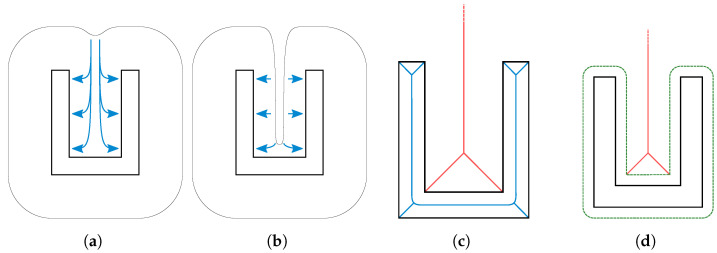
As the optimization process adapts temperatures on the offset surface, long distances between the offset surface and the part surface as shown in (**a**) lead to suboptimal optimization results. It is more desirable to have offset surfaces closer to the part surface as shown in (**b**). This is especially problematic in concave regions as shown here. Outer parts of the medial axis (**c**, red) are used to estimate regions into which the offset may not extend. The medial axis is clamped by a minimal distance to the part surface (**d**) to assure that the decrease in optimization is not too prohibitive.

The input to this work’s algorithm is a triangle surface mesh of the workpiece. The goal is to generate an offset surface mesh that defines the optimization domain for the computation, as shown in Section 2.1. It has already been established in Section 4 that there are two distinct target goals:

- The offset distance from the surface depends on the thickness of the local part geometry.
- Offsets should not connect at concave surfaces, as shown in Figure 10, to offer the optimization algorithm better control over temperatures closer to the part surface.

This work’s algorithm relies on a discrete volumetric, i.e., voxel-based intermediate representation. This allows trading precision for simplicity and algorithmic stability. The resulting offset is not required to be highly precise as it does not represent exact part geometry but only limits the optimization domain.

**Figure 11:**
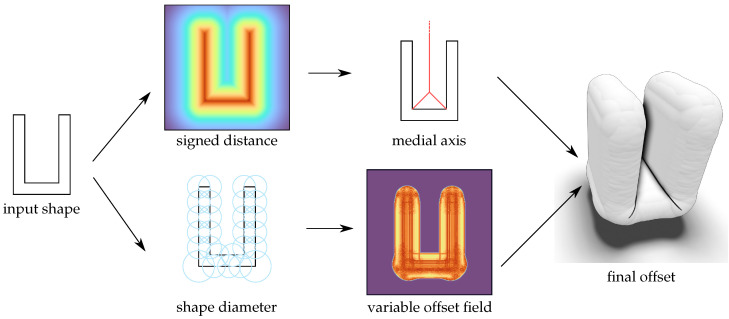
Overview of this work’s pipeline: Given an input shape, a signed distance field is captured, and the medial axis outside the input is extracted. Additionally, a shape diameter function of the input is estimated from which a variable offset field is then computed. By combining the medial axis and the variable offset into a single field, the final variable offset mesh can be extracted.

The overall pipeline is shown in Figure 11. From the input shape, two intermediates are computed. For the variable offset (Figure 11, bottom), a *shape diameter function* [37] is determined. Then, this is mapped to a set of rasterized offset spheres in the same resolution as the voxel grid. Afterwards, the signed distance function of the spheres near the surface is efficiently evaluated to enable extraction of an offset surface using *marching cubes* [31]. However, to avoid the coalescence of the offset surface in concave regions of the geometry, the medial axis of the input is computed input using the signed distance function toward the input shape (Figure 11, top). Then, this is combined with the variable offset field before isosurface extraction.

### 5.1. Shape Diameter Function

In Section 4.2, it was established that the offset computation should depend on the local thickness of the input shape. Thicker parts of the input achieve an optimal temperature distribution for low offset thicknesses, whereas 1 to 2 mm seem to have a local optimum for higher thicknesses. Considering that the resulting offset is the search space for the regions in which cooling canals can be planned, it must depend on the local thickness of the input surface. Therefore, the radius of the offset spheres depends on the local thickness of the input. This *thickness* can be computed using a *Shape Diameter Function*. For this, a method adapted from [37] is employed. A set of sample positions on the input surface is defined where the diameter should be determined additionally with a set of corresponding inward-pointing vectors within a cone around the normal. This results in a set of query rays that are used to define where the diameter should be evaluated. To generate the samples, the input mesh is rasterized in the *x*, *y*, and *z* direction using the same resolution that is later used for the efficient offset field extraction in Section 5.3 assuring that each surface voxel corresponds to at least one thickness value.

To avoid issues with numerical rounding imprecision, each ray origin is shifted by an ϵ in inwards normal directions. Then, for each query ray, a random set of rays in a cone surrounding the query ray is generated. For each ray, the distance to the opposite side of the input surface is evaluated. Then, the diameter is given by first median-filtering the resulting distances and then averaging the remaining ones. As the diameter is quite noisy, a Gaussian smoothing is additionally applied to the diameters in the local neighborhood of each query point.

### 5.2. Medial Axis

As shown in Section 4.3, it is beneficial for the thermal optimization process if the offsets of separate concave elements are not allowed to merge. One way to prevent this is by disallowing the offset to grow across surfaces defined by the *medial axis*. The medial axis is defined as those points that have the same shortest distance to at least two distinct points of the input surface.

While there is an active field of research for determining the medial axis as precisely as possible [38], it suffices in our applications if an approximation is given that can be represented in the voxel grid. Similarly to Xia et al. [39], the medial axis is estimated by evaluating the Laplacian of the distance transform of the input solid. Then, getting a coarse approximation is achieved by thresholding the Laplacian with a small negative ϵ.

**Figure 12:**
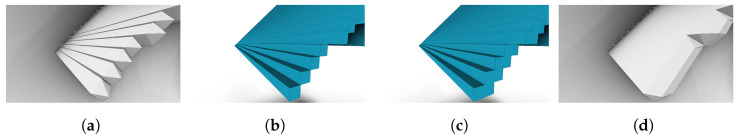
Undesired, fork-like artifacts after isosurface extraction (**a**) are caused by thin, diagonal (edge connected) layers in the voxel grid of the medial axis (**b**). To avoid this, it is assured that neighboring voxels are instead face-connected (**c**), which results in a much cleaner output offset (**d**).

**Figure 13:**
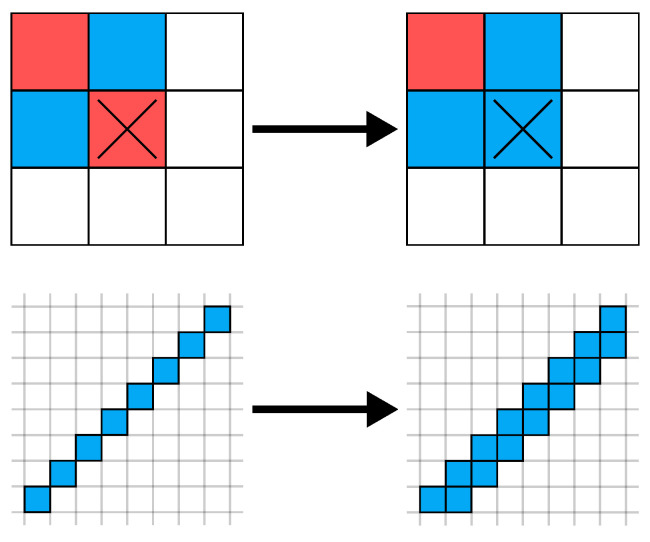
The *stair-connect* morphological operation: To eliminate fork-like artifacts during isosurface extraction, it is assured that all stair-like components are not only connected diagonally but also by direct neighborhood. This operation is applied once for each axis direction and both for rising and falling stairs, resulting in six total applications.

Unfortunately, using a fixed threshold results in noisy regions of the medial axis. This is undesired during isosurface extraction, as it results in *fork-like* artifacts, as shown in Figure 12 that are difficult for the thermal optimizer. They are eliminated using a range of morphological operations [40]. First, three iterations of each dilation and erosion are applied to close small holes. Then, all staircase artifacts are eliminated by using a 3×3×3 filter in positive directions, as shown in Figure 13.

In order to guarantee physical integrity, only those parts of the medial axis that have a minimum distance from the input surface are included. The reasoning here is that cooling canals cannot lie arbitrarily close to the part geometry due to physical constraints. This means that choosing a minimal offset that lies too close to the input surface would prohibit the generation of cooling channels close to the surface in these regions, as they can only lie inside the offset volume. By limiting how close the medial axis cutout is allowed to the input, it is assured that a minimal offset is maintained for these regions.

### 5.3. Efficient Offset Extraction

**Figure 14:**
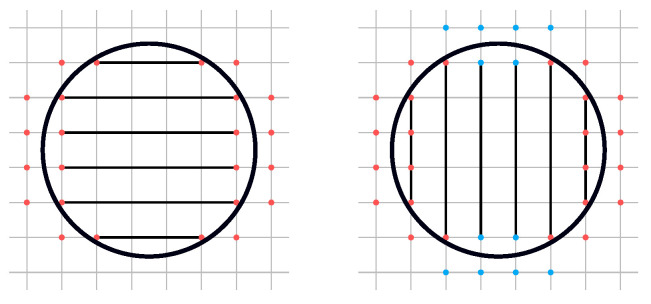
To efficiently extract the variable offset, each offset sphere is rasterized in the *xyz* direction, and the signed distance to the sphere is evaluated at the two grid points closest to the surface in the rasterization direction. On the left is shown where the signed distance is evaluated when rasterizing in the *x* direction, which is followed by a rasterization step in the *y* direction on the right, resulting in the blue samples. The *z* direction is handled analogously.

After having computed the shape diameter function (Section 5.1) and the medial axis (Section 5.2), they can be combined to extract a boundary mesh with variable offset. The process is shown in Figure 14. In the first step, an approximate signed distance field is computed that is only exact near the surface of the desired variable offset surface. Non-exact samples need only correctly classify inside from outside. By convention, negative values are defined to be inside the shape, while positive values are outside.

Initially, a sphere is defined for each sample point and its corresponding radius. Then, the output offset is defined as the envelope obtained from the union of all spheres. Each sphere is rasterized in *xyz* directions separately using the same resolution as the output voxel grid (Figure 14). This reduces the complexity of rasterizing a single sphere from $O(n^{3})$ to $O(n^{2})$. Each rasterization ray *r* results in two intersection points $x_{1}$ and $x_{2}$ with the sphere. Along *r*, the four grid points $s_{i}$ are determined for which the signed distance function to the sphere should be evaluated by rounding the depth component up and down to the nearest integer value. Then, the grid is updated for each sampled signed distance if the new value is smaller than the previously stored value. This assures that only voxels near the sphere surface are evaluated. After having rasterized each sphere in all three axes’ directions, the grid is guaranteed to have correct signed distance functions close to the surface. However, there may still be uninitialized grid nodes that must be classified as inside or outside to assure that the isosurface extraction produces a correct result. This is solved by applying a flood filling starting from one of the corners of the grid for which it is known that it has to be outside the offset surface. All connected grid nodes are marked as outside by setting their distance value to a positive value, i.e., infinity. Then, all remaining uninitialized cells must be inside and are set to negative infinity.

To incorporate the medial axis, all voxels that are part of the medial axis are overwritten to be outside. Using the resulting voxel grid, an isosurface at the zero crossing is extracted.

### 5.4. Post-Processing

As the output mesh is generated from a regular grid using marching cubes, the output complexity, i.e., the number of triangles, is much higher than necessary. Especially planar regions will generate triangle counts that are quadratically proportional to the voxel resolution. Higher-than-necessary triangle counts are undesired, as they increase the problem complexity for the thermal optimizer. Therefore, a simple mesh decimation [41] is applied to reduce the output complexity while limiting the output error, which is followed by an iteration of Laplacian smoothing to improve the surface quality.

## 6. Evaluation

### 6.1. Performance

**Figure 15:**
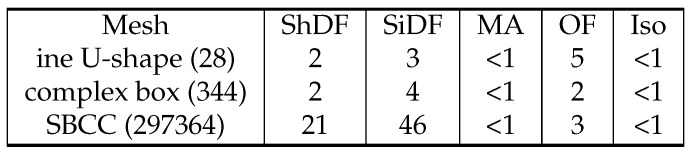
The time this work’s algorithm spent in each step (Shape Diameter Function (ShDF), Signed Distance Function (SiDF), Medial Axis (MA), Offset Field (OF), isosurface extraction (Iso)) in seconds for various inputs. The number next to the model name is the number of triangles.

The developed algorithm is implemented using C++17 and compiled with Clang 7. All benchmarks are done on a machine with an Intel(R) Core(TM) i7-6700K CPU that runs at 4 GHz and has 32 GB RAM. The timings for different inputs are shown in Figure 15. Even though the computation of the *shape-diameter-function* (Section 5.1) and the *signed-distance-function* that is precursor for the medial-axis computation (Section 5.2) scale with the number of input triangles, the total computation time, even for complex models such as the SBC case taking a little over a minute, are miniscule when compared to the time spent optimizing the thermal distribution (Section 2.1) that can take hours or even days. The time saving is also significant compared to the conventionally manual design of the offset, and it scales better with the complexity of the part’s geometry.

### 6.2. Qualitative Results

**Figure 16:**
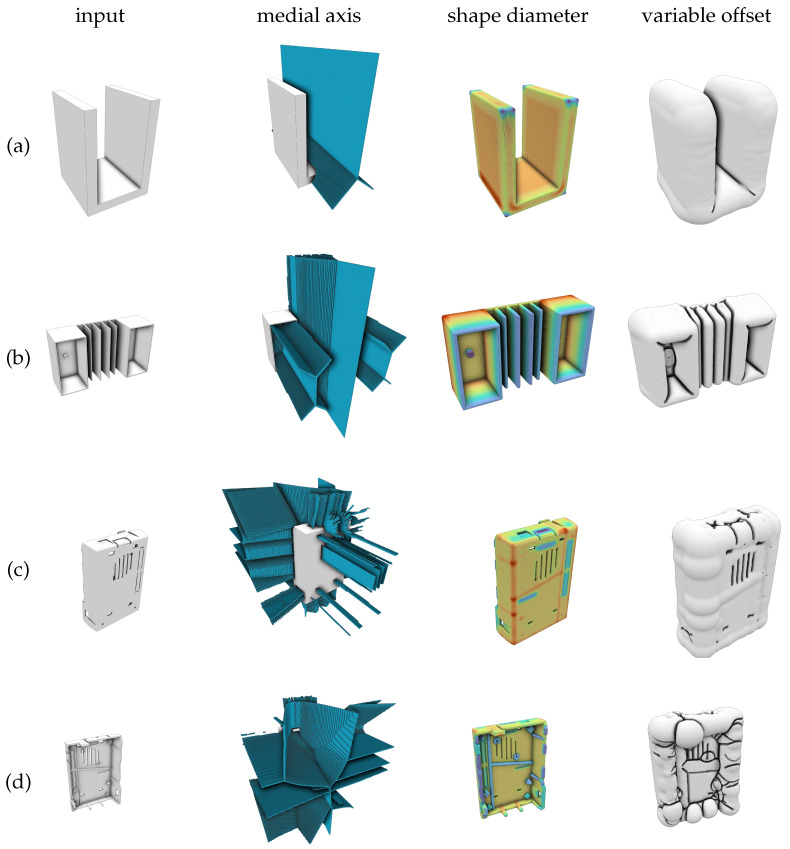
A selection of sample inputs during each processing stage. For each input, its medial axis, shape diameter, and the final variable offset are shown. The inputs are (**a**) a simple U-shape, (**b**) a more complex box, front (**c**) and back (**d**) of a single board computer case (SBCC).

The pipeline is tested on several inputs. A selection is shown in Figure 16. As a volumetric approach is applied, the offset computation is independent of the triangulation of the input meshes and works equally well for various inputs. For the shown samples, a voxel resolution of 256 is used, which is found sufficient for this use-case. During testing, voxel resolutions up to 512 are tried. While higher resolutions offer more accurate offsets, they also produce much more triangles during isosurface extraction that have to be eliminated in the post-process. Unfortunately, the loading time of the used optimization software *Comsol Multiphysics* was found to increase drastically with higher triangles counts. Therefore, the number of output triangles is reduced during post-processing (see Section 5.4).

### 6.3. Validation of the Automatically Generated Offset in Thermal Optimization

**Figure 17:**
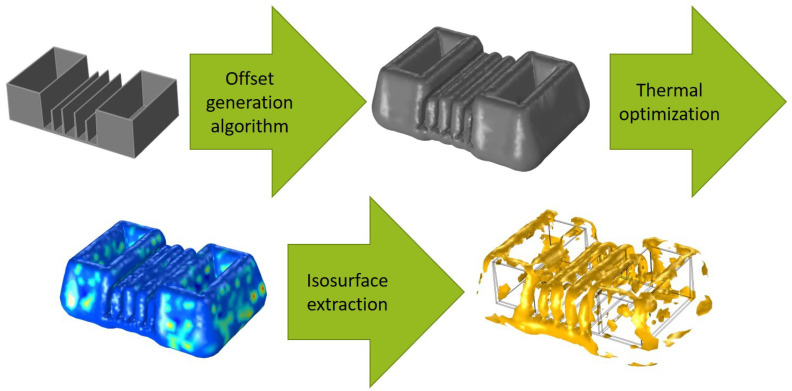
Application of the developed algorithm for an automized and optimized offset generation and the concluding thermal optimization.

The generated offset is imported to the thermal optimization model, and a solid geometry is derived from the triangulated surface. This step is necessary to create geometric boundaries that can be selected for the definition of the materials, evaluation areas, and surface for the optimization parameters. An insufficient description of the offset results in defective geometry. The next step is a new mesh of the offset geometry. Accordingly, no surface elements from the input file are used. Figure 17 shows the application of the offset generation algorithm and the successful execution of thermal optimization. The resulting isosurface for a later cooling channel simulation depicts a reasonable distribution. Additionally, the usage of the medial axis for an increased optimization surface shows better results. The quality function (see Equation (1)) gives 0.167841 when the medial axis is calculated and removed, whereas offsets without additional surface give 0.295934. This decrease of 43% suggests a better overall cooling, and less warpage is expected.

## 7. Conclusions

This work shows the necessity of an automized generation of the contour. The manual design of the offset is not feasible, especially for complex parts such as the raspberry pi case. Additionally, the influence of the variation occurring in manual work needs to be avoided for a reproducible and comparable offset generation.

Multiple steps have been identified as variables that have an influence on the thermal optimization result. For one, the offset thickness in combination with the wall thickness of the part has a significant influence on the optimal temperature distribution. The influence of the cooling rate and freeze time on the second part of the quality function will be investigated with more practical dimensions of the geometry. Additionally, a solution for the coalescence of concave geometries and therefore multiplication of the effective offset distance is determined. The calculation and extraction of the medial axis pose as a suitable method to establish fitting offset thicknesses in cavities. For the first tests with one geometry containing a slit at the medial axis, a multiple times better result for the quality function is achieved. In future work, it needs to be determined, whether the gap in the thermal model at the medial axis leads to impossible temperature fields, which cannot be approximated by actual cooling channels. In this case, adaptations and limitations of the extracted medial axis will be considered.

The variable offset generation has been successfully implemented into an interactive tool that can be used for further experiments. The developed algorithm considers the examined effects, and the generated offsets can be used in the existing methodology. The first results show a significant improvement of the optimized thermal balance in the mold.

## Data Availability

Not applicable.
